# Nanomaterial-Based Therapeutic Delivery: Integrating Redox Biology, Genetic Engineering, and Imaging-Guided Treatment

**DOI:** 10.3390/antiox15040430

**Published:** 2026-03-30

**Authors:** Dorota Bartusik-Aebisher, Daniel Roshan Justin Raj, David Aebisher

**Affiliations:** 1 Department of Biochemistry and General Chemistry, Collegium Medicum, Faculty of Medicine, University of Rzeszow, 35-310 Rzeszow, Poland; dbartusikaebisher@ur.edu.pl; 2 English Division Science Club, Collegium Medicum, Faculty of Medicine, University of Rzeszow, 35-310 Rzeszow, Poland; dj135639@stud.ur.edu.pl; 3 Department of Photomedicine and Physical Chemistry, Collegium Medicum, Faculty of Medicine, University of Rzeszow, 35-310 Rzeszow, Poland

**Keywords:** nanomaterials, drug delivery systems, targeted drug delivery, gene delivery, theranostic nanoparticles

## Abstract

Nanomaterials are emerging versatile platforms for therapeutic delivery, as they offer precise control over drug, antioxidant, and genetic payload transport across biological barriers. Inorganic, organic, hybrid, and biomimetic systems are the major classes of nanomaterials, which all have different physicochemical properties such as size, surface charge, and surface functionalization. These properties collectively influence stability, biodistribution, cellular uptake, and release kinetics. Engineering strategies are increasingly using stimuli-responsive designs that are triggered by pH, reactive oxygen species (ROS), and intracellular redox gradients to perform spatially and temporally controlled delivery. Antioxidant and redox-modulating nanocarriers are of great importance as they overcome the limited bioavailability and nonspecific activity of conventional antioxidants by improving stability, targeting oxidative microenvironments, and allowing for regulated release. Improvements in lipid, polymeric, and inorganic nanoplatforms have also developed gene delivery applications, including siRNA, mRNA, and CRISPR/Cas systems, to provide better cytosolic release and precise therapeutics. When diagnostic imaging is integrated with therapy through theranostic nanoparticles, real-time monitoring and personalized intervention are possible. Safety, scalable manufacturing, and regulatory alignment are some challenges that show the need for standardization and translational procedures to utilize the potential of theranostic nanomedicine.

## 1. Introduction

Nanomaterials in the world of modern biomedicine are finely engineered structures that typically range from 1 to 100 nm in size and can act as drugs, imaging agents, or most commonly as delivery vehicles, which are controlled by nanoscale interfacial effects [1]. A practical classification that is used in nanomedicine groups existing systems into lipid-based carriers, polymeric nanocarriers, dendrimers, and inorganic/metal or carbon-based nanomaterials, with “hybrid” designs becoming more common [2,3]. Historically, the development of the sector was accelerated when liposomal and polymer-based systems had become clinically validated products, exemplified by PEGylated liposomal doxorubicin, also known as “Doxil^®^” or “Caelyx^®^,” which was approved in 1995 and is often cited as the first FDA-approved nanodrug [4]. The amount and diversity of approved and clinical-stage nanopharmaceuticals have grown since the 1980s–1990s, spanning across various fields such as oncology, imaging, and much more, as seen in surveys of marketed nanomedicines [5]. Their small size is a key physiochemical property that controls circulation, biodistribution, clearance, and tissue penetration while also providing rationale for the accumulation of tumors passively through the enhanced permeability and retention (EPR) effect [6,7]. Surface charge, which is often quantified as zero potential, strongly influences the formation of protein corona, stability of colloids, and cell interactions, with both experimental and review literature linking charge to its uptake mechanisms and fate in vivo [8,9]. Surface functionalization, which is PEGylation for “stealth” and ligands such as antibodies and peptides for active targeting, is considered an important design lever to improve biocompatibility, circulation time, and receptor-mediated delivery, allowed by modern bioconjugation toolkits [10,11,12]. Phase engineering, heterostructure designs, and highly advanced surface modifications are emerging strategies that can further improve the redox-related performance of nanomaterials. Crystal structure and defect density can be tuned with phase engineering which will influence the electron transfer and ROS catalytic activity. Heterostructures can improve the overall efficiency of redox cycling by promoting interfacial charge separation. Defect engineering and functional coatings are some surface modification techniques that are able to improve electron transfer pathways and reactive oxygen species (ROS) interactions. This will determine antioxidant or pro-oxidant behavior. In general, these approaches give a mechanistic basis for parameters such as stability, catalytic activity, and therapeutic specificity in redox-responsive nanomedicines [13].

Reactive oxygen species (ROS) can sometimes be a “double-edged” signaling molecule, as moderate increases can drive redox signaling that supports the proliferation, survival, angiogenesis, and metastasis of many types of cancers, while excessive amounts of ROS can trigger the death of tumor cells [14]. Oxidative stress in neurodegeneration, for example, damages lipids, proteins, and nucleic acids and is linked to Alzheimer’s disease and other disorders with increasing brain vulnerability due to high oxygen use and relatively limited antioxidant defenses [15]. ROS from sources such as NADPH oxidases, mitochondria, and endothelial dysfunction in cardiovascular diseases contribute to atherosclerosis, remodeling, arrhythmias, and the progression of heart failure [16]. Inflammatory signaling is also very closely associated with redox biology, as ROS are able to activate or repress nuclear factor-kappa B (NF-κB) in some contexts, and NF-κB can also induce pro-oxidant genes, which further adds to inflammation-oxidation stress loops [17,18]. These roles have made oxidative stress an attractive therapeutic target, but “blanket” antioxidant scavenging has struggled due to ROS being compartmentalized, short-lived, and also being required for normal signaling and host defense [19]. Conventional supplementation trials have often shown null or even harmful outcomes, including the meta-analytic signals of increased mortality with a higher dose of beta-carotene and vitamin E in some settings [20]. A key limitation of antioxidant therapy is the “antioxidant paradox,” that is, when excessive or misplaced antioxidants can cause reductive (antioxidative) stress or even fail to reach the relevant ROS source, which limits their effectiveness. These limitations have caused a shift in therapeutic strategies toward targeting the production of ROS at its source, such as inhibiting enzymatic generators like NADPH oxidases (NOX), rather than neutralizing reactive species indiscriminately throughout the cell [21].

Many antioxidants, including polyphenols, have been limited clinically by factors such as poor aqueous visibility, rapid metabolism, and chemical photosensitivity. Because of this, many nanocarriers are widely being used to improve bioavailability and stability by protecting unstable molecules from degradation and also by enhancing apparent solubility and absorption [22]. This rationale is demonstrated very well for curcumin, where some formulation strategies, including nanoparticles, have been repeatedly linked to a higher systemic exposure and better performance than the free compound [23], and also in resveratrol, where more importance has been placed on nanoencapsulation in order to be protected from instability and improve delivery [24]. Beyond exposure, nanomaterials allow for controlled release and reduced systemic toxicity by way of tuning the composition and morphology of carriers to regulate release kinetics and maintain therapeutic levels while limiting adverse effects related to peaks [25]. Targeted nanoparticle delivery is also often referred to as a route to better efficacy with a lower off-target burden [26], while safety-focused reviews have highlighted that nanotoxicity must be evaluated alongside these benefits [27]. As many antioxidants are potent in vitro but exposure is limited in vivo, nanodelivery addresses the main translational issues by getting stable, sufficient concentrations to the correct tissues over time [28].

In recent times, nanomaterial-based drug delivery, antioxidant nanomedicine, gene delivery systems, and theranostic platforms have studied individually in many reviews. These topics are however discussed in isolation and there is a lack of integration between redox biology and the design of nanomaterials for application. In this review, redox-responsive nanomaterials are taken into perspective as the central mechanistic framework. It will also study the design, functional engineering, and translational potential of nanomaterials for therapeutic delivery, with more focus put on antioxidant, redox-responsive genetic applications. It discusses the advances in targeted drug delivery, gene therapeutics, and theranostic nanoplatforms while also critically evaluating the pharmacokinetics, safety considerations, and translational challenges that are involved with the future of precision nanomedicine. By forming a connection between these domains, this review will provide a framework that is more integrated and translationally relevant in comparison with existing literature.

To guide the reader through this review, the following sections have been structured so that they progressively build on the fundamental principles to translational applications. Section 2 provides an outline of the classification of nanomaterials and their physicochemical properties that control biological interactions. Section 3 discusses the design and engineering behind nanomaterial-based drug delivery with an emphasis on redox-modulating therapies. Section 4 focuses on the various strategies used in targeted delivery including active, passive, and redox-responsive approaches. Section 5 examines how nanomaterial platforms are used for gene delivery, highlighting the intracellular barrier and redox-triggered release mechanisms. Section 6 presents imaging and therapy that can be integrated through theranostic nanoparticles. And finally, Section 7 addresses the safety and regulatory challenges as well as the field’s future directions.

**Figure 1:**
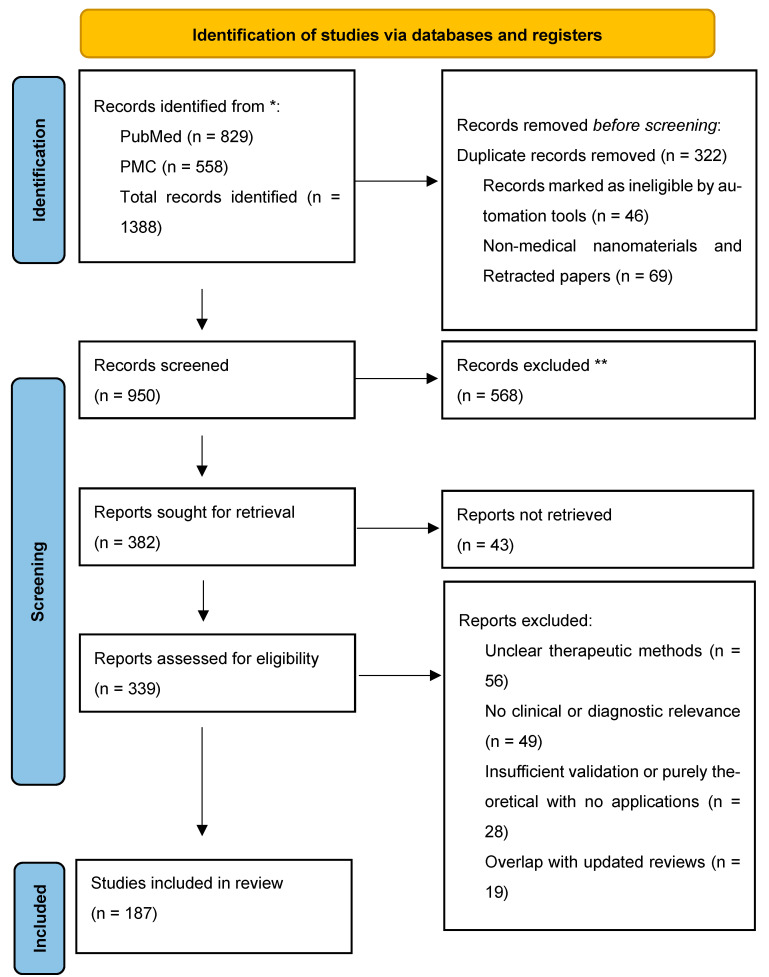
Preferred Reporting Items for Systematic Reviews and Meta-Analyses (PRISMA) flow diagram that represents the methods behind the selection of literature included in the review. The initial record is marked with (*) and excluded record is marked with (**).

This review is in accordance with PRISMA guidelines to ensure a transparent and systemic selection of studies, which is visualized in Figure 1. A comprehensive search of the PubMed and PubMed Central (PMC) databases provided 1388 records. After the removal of duplicates (n = 322), marked ineligible (n = 46), and non-medical or retracted publications (n = 69), 950 records were further screened. Following abstract screening, 382 reports were sought for full-text retrieval, out of which 339 were assessed for eligibility. Studies were then further excluded for their unclear therapeutic methodology, the lack of clinical or diagnostic relevance, insufficient validation, or overlap with updated reviews. In the final analysis, 187 studies met the inclusion criteria and were incorporated in the qualitative synthesis. Beyond these methodological criteria, more importance was placed on the studies that had provided mechanistic insight into nano-bio interactions and redox-responsive or disease-targeted functionality. Their relevance to clinically translatable delivery systems is key, as it guides the thematic focus of the review.

## 2. Nanomaterials for Biomedical Therapeutic Delivery

### 2.1. Types of Nanomaterials

Nanomaterials that are used in biomedical and pharmaceutical research are commonly classified as inorganic, organic, and hybrid or biomimetic systems, which is according to the various differences in their composition, physicochemical behavior, and biological interactions. The material classes that are discusses in this section were chosen based on their established roles in therapeutic delivery and also because they can be rationally engineered for redox modulation, targeting, controlled release and other functions. Although there are many other emerging nanomaterials, the systems discussed here have the strongest experimental validation and translational relevance. Inorganic nanomaterials include gold, iron oxide, silica, and ceria nanoparticles, with each of them offering unique functional advantages as seen in Table 1. Gold nanoparticles have chemically stable cores and highly tunable surfaces, which are favorable and allow for conjugation with drugs, biomolecules, or targeting ligands while also allowing for optical and photothermal applications due to localized surface plasmon resonance [29]. Iron oxide nanoparticles have superparamagnetic properties that allow for magnetic resonance imaging contrast, magnetic targeting, and therapies based on hyperthermia, with surface coatings playing an important role in determining biocompatibility and circulation behavior [30]. Silica nanoparticles, particularly mesoporous silica systems, possess high surface area and tunable pore architectures, which help make drug loading and stimuli-responsive release efficient while also allowing for extensive surface functionalization [31]. A unique inorganic nanomaterial is cerium oxide because of its reversible redox cycling between Ce^3+^ and Ce^4+^ states, providing enzyme-mimetic antioxidant behavior that is strongly influenced by the surrounding biological environment [32]. In contrast, organic nanomaterials are usually composed of lipids or polymers and are often designed for biodegradability and long-term toxicity reduction. Lipid nanoparticles have become the leading platforms for nucleic acid delivery, as they are able to encapsulate and protect RNA and DNA, assist with cellular uptake, and promote endosomal escape through carefully balanced lipid compositions [33]. Polymeric nanoparticles, including systems that are based on biodegradable polymers such as poly lactic-co-glycolic acid (PLGA) or PEG-modified architectures, allow for controlled and sustained drug release while also providing flexibility in size, surface chemistry, and payload compatibility [34]. A structurally precise subclass of organic nanomaterials is dendrimers, which are characterized by branched architectures and multivalent surfaces that allow for high-density functionalization and drug conjugation, even though their biological performance is strongly influenced by the surface charge and generation number [35]. Combining inorganic and organic categories together are the hybrid and biomimetic nanomaterials, which connect multiple components to provide stability, functionality, and biological compatibility. This strategy is illustrated effectively on lipid–polymer hybrid nanoparticles by integrating polymeric cores with lipid shells to obtain robust structures and ideal biointerfaces [2]. Biomimetic nanomaterials such as cell-membrane-coated nanoparticles build on this concept even more by using natural cellular membranes to mask synthetic cores, which allows for immune evasion, prolonged circulation, and biologically relevant targeting while at the same time causing new challenges in manufacturing consistency and large-scale clinical implementation [36].

### 2.2. Physicochemical Properties Relevant to Delivery

**Figure 2:**
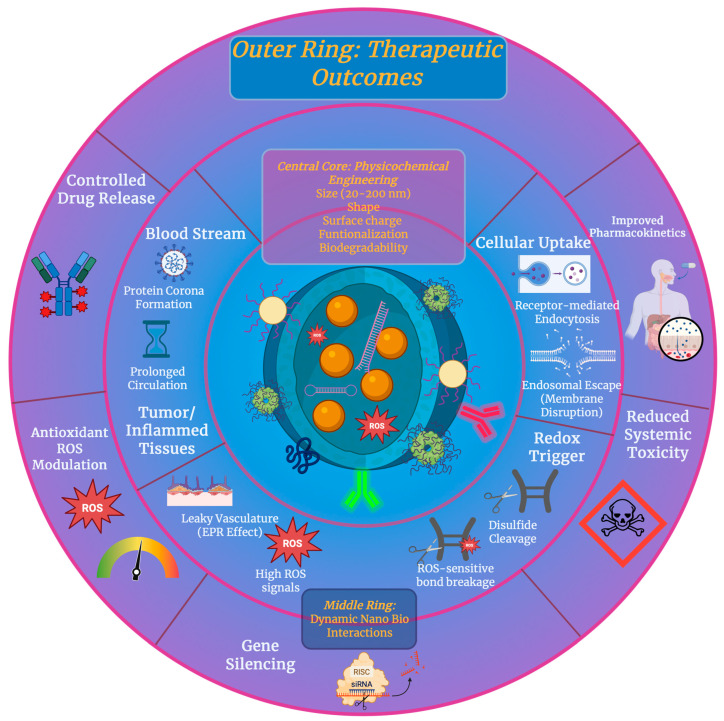
A conceptual system that illustrates the precise engineering behind redox-responsive nanomaterials for biomedical therapeutic delivery. The central core represents physicochemical design parameters such as size, charge, functionalization, and biodegradability which are responsible for nano-bio interactions including stability during circulation, targeting, cellular uptake, and redox-triggered release. These processes ultimately support controlled payload delivery, oxidative stress modulation, and improved therapeutic outcomes. Created in BioRender. Roshan, D. (2026) https://BioRender.com/jgcr2ep, accessed on 21 March 2026.

The efficiency and safety of nano- and micro-scale delivery systems are largely based on a defined set of physicochemical properties, which include size, shape, surface charge, surface functionalization including redox responsiveness, and biocompatibility with controlled biodegradability. Together, these parameters influence the stability of colloids in biological fluids, biodistribution, cellular uptake, intracellular trafficking, and payload release. Most importantly, they function as an interdependent system rather than as isolated variables [37,38]. Particle size is a huge factor that controls circulation behavior and cellular internalization. Numerous studies have reported the optimal uptake of nanoparticles in non-phagocytic cells that range around tens of nanometers, which shows a balance between membrane-wrapping energetics and endocytic efficiency. Size also determines the clearance pathways of particles, with smaller particles undergoing renal elimination and larger ones by the uptake of the mononuclear phagocyte system [39]. Shape is another layer of control, where non-spherical carriers such as rods and disks show an altered margination in blood flow, different adhesion to endothelial surfaces, and distinct uptake kinetics in comparison with spherical particles. Differences that are shape-dependent come from changes in contact area and membrane deformation during internalization [40]. Surface charge affects interactions with cell membranes, protein corona formation, and colloidal stability. Enhanced cellular uptake is often seen in positively charged carriers due to the electrostatic attraction to negatively charged membranes, but they are also related to an increase in cytotoxicity and nonspecific interactions. Neutral or slightly negative surfaces usually reduce unwanted protein absorption and prolong circulation; however, to ensure an efficient uptake, they may require more targeting strategies [37]. Surface functionalization is used when it is needed to tune biological interactions by introducing stealth properties, targeting ligands, or stimuli-responsive elements. Redox-responsive systems are particularly relevant as intercellular environments, and many pathological tissues are significantly different in redox state from blood plasma. By integrating disulfide bonds into carrier backbones or crosslinks, the rise in intracellular glutathione levels had been utilized, which enables extracellular stability followed by rapid intracellular degradation and cargo release, which is illustrated in Figure 2 [41,42]. On top of reduction-sensitive designs, oxidation-responsive motifs that target ROS have also been developed, supporting selective release in inflamed or tumor tissues characterized by oxidative stress. Redox-triggered strategies improve spatial and temporal control over delivery and reduce premature release [43]. Biocompatibility and biodegradability are crucial factors in clinical translation, meaning that materials must be able to degrade into non-toxic byproducts at a controlled rate while avoiding long-term accumulation or immune activation. A good example where this can be seen is in PLGA, where it degrades into lactic and glycolic acids that enter natural metabolic pathways. It is possible to tune its degradation kinetics and release profiles through factors such as polymer composition and molecular weight [44]. Overall, a well-designed delivery system requires an excellent optimization of physicochemical properties.

**Table 1:** Overview of major nanomaterial classes used for biomedical therapeutic delivery and their key properties.

Representative Materials	Nanomaterial Class	Key Physicochemical Features	Main Functional Advantages for Delivery	Limitations/Challenges	Typical Therapeutic Applications
Gold nanoparticles [29]	Inorganic	Chemically inert core, easily tunable surface chemistry, strong plasmonic/optical response	Straightforward conjugation of drugs and targeting ligands, allowing photothermal and optical theranostics	Non-biodegradable core, risk of long-term tissue accumulation	Targeted drug delivery, photothermal cancer therapy, optical imaging
Iron oxide nanoparticles [30]	Inorganic	Superparamagnetic behavior, magnetic field responsiveness	MRI contrast, magnetic targeting, and magnetically induced hyperthermia	Rapid uptake by mononuclear phagocyte system without stealth coatings	Image-guided therapy, magnetically targeted delivery
Mesoporous silica nanoparticles [31]	Inorganic	Very high surface area, tunable pore size and volume, robust framework	High drug loading and stimuli-responsive release; extensive surface functionalization	Relatively slow biodegradation, potential silica-related toxicity at high doses	Controlled and triggered release of small molecules and biomacromolecules
Cerium oxide nanoparticles [32]	Inorganic (redox-active)	Reversible Ce^3+^/Ce^4+^ redox cycling, catalytic ROS scavenging	Enzyme-mimetic, self-regenerating antioxidant and redox-modulating activity	Biological effects strongly depend on local environment and surface chemistry	Antioxidant and anti-inflammatory therapies
Lipid nanoparticles (LNPs) [33]	Organic (lipid-based)	Amphiphilic, self-assembled lipid structures, encapsulate nucleic acids	Protect and deliver RNA/DNA, promote cellular uptake and endosomal escape, biodegradable	Formulation and storage stability issues; possible innate immune activation	Messenger RNA (mRNA)/small interfering RNA (siRNA) and gene delivery
PLGA and PEGylated polymeric nanoparticles [34,44]	Organic (polymeric)	Biodegradable polymers with tunable molecular weight and composition	Controlled and sustained drug release, adaptable size and surface chemistry	Burst release and local acidification during degradation is possible	Long-acting small-molecule and protein delivery
Dendrimers [35]	Organic (dendritic)	Highly branched, monodisperse, multivalent architecture	High-density attachment of drugs/ligands, precise structural control	Surface charge–dependent cytotoxicity, complex and costly synthesis	Targeted drug and gene conjugate delivery
Lipid–polymer hybrid nanoparticles [2]	Hybrid	Polymeric core with lipid shell combining rigidity and biomimetic interface	Improved structural stability with favorable biointerface and circulation	More complex and less scalable manufacturing	Multifunctional and combination drug delivery
Cell membrane–coated nanoparticles [36]	Biomimetic	Natural cell membrane proteins and lipids cloaking a synthetic core	Immune evasion, prolonged circulation, and biologically relevant targeting	Batch variability and scale-up challenges	Targeted therapy for cancer and inflammatory diseases

**Figure 3:**
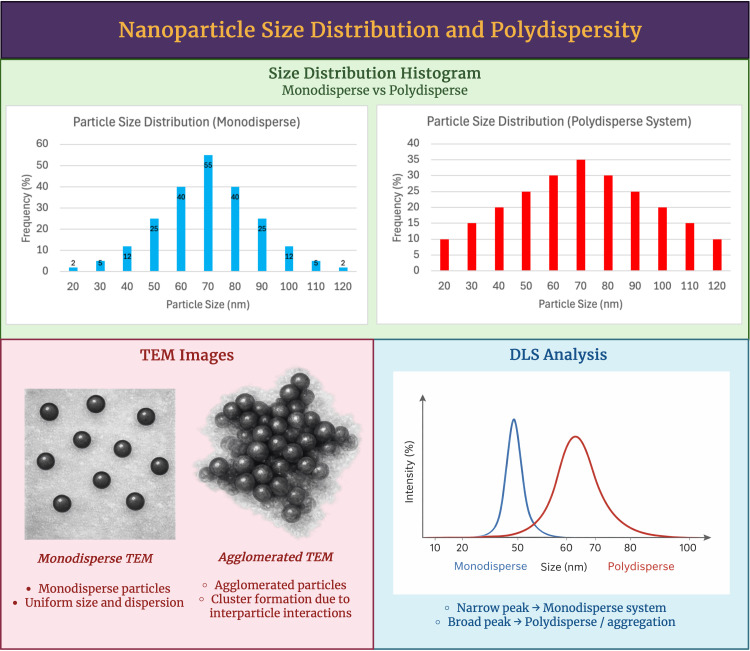
Particle size distribution histograms show the monodisperse and polydisperse populations which depicts the differences between synthesis uniformity and heterogeneity. The schematic TEM images reveal the difference in the morphology of the particles in monodisperse and agglomeration. DLS intensity distribution curves show the hydrodynamic size profiles, where narrow peaks reflect the homogeneous populations and broader peaks indicate an increase in polydispersity and potential aggregation. The figure serves as a concept and does not represent any experimental data. Created in BioRender. Roshan, D. (2026) https://BioRender.com/02yff1z, accessed on 21 March 2026.

A technique that is largely used to describe the surface chemistry of nanoparticles is the Fourier-transform infrared (FTIR) spectroscopy. It can also be used particularly in the phytochemical-assisted synthesis to confirm the presence of capping agents [45]. On top of the basic functional group identifications, evidence of nanoparticle and biomolecule interactions can be seen in the characteristic band shifts such as hydroxyl (–OH), carbonyl (C=O), and aromatic (C=C) groups. The changes in peak position, intensity, or broadening that is relative to the precursor extracts may indicate a metal-ligand coordination between various phytochemicals such as polyphenols and flavonoids, and the surface of the nanoparticle. These spectral features support the presence of phytochemical capping [46]. This can improve colloidal stability, reduce agglomeration, and influence the biological interactions. Thus, FTIR analysis is not only a qualitative tool that is being used in functional group assignment but also serves a purpose of being a mechanistic indicator of surface functionalization [47]. It is important to not only consider the size of the average particle but also the particle size distribution as that will be vital to better describe the morphology of the nanomaterial. This is usually shown in a histogram such as the one in Figure 3, because it can depict the population heterogeneity and polydispersity [48]. Uniform synthesis and improved stability can be seen in narrow distributions. Whereas broader distributions can suggest a presence of secondary particle populations and variability. Additionally, agglomeration effects should also be considered as the interparticle interactions and insufficient surface stabilization are factors that may lead to clustering in solution. This can show larger hydrodynamic sizes when compared to the primary particle dimensions that were observed by microscopy. Such an aggregation behavior has potential to significantly influence the stability of colloids, cellular uptake, and biodistribution [49]. Therefore, it is considered as a crucial parameter while evaluating the performance of nanomaterials.

## 3. Nanomaterial-Based Drug Delivery Systems

### 3.1. Design and Engineering of Drug Delivery Systems

Nanomaterial-based drug delivery systems (DDS) are designed to encapsulate certain therapeutic molecules and release them at the desired targeted sites in a controlled and stimuli-responsive manner, which will help overcome some challenges surrounding conventional drugs, such as poor solubility, rapid clearance, and systemic toxicity. This section focuses on nanomaterial systems that have design principles that are directly related to therapeutic performance, particularly in redox sensitivity and controlled release behavior. Systems that do not have a clear linkage between the properties of materials and delivery outcomes have not been emphasized. Table 2 discusses various different types of DDS and their functional engineering strategies. These systems are engineered to have control over drug encapsulation and conjugation strategies, as well as the integration of stimuli-responsive functions that utilize pathological microenvironments for on-demand drug release. Attaining efficient drug encapsulation and conjugation within nanoscale carriers while also preserving the activity and stability of the drug remains a design challenge in DDS engineering. Therapeutic agents can be incorporated through physical encapsulation or chemical linkage in nanocarriers such as polymeric nanoparticles, liposomes, and prodrug conjugates. Chemical conjugation, which is often via acid-labile bonds or polymer-drug linkages, is known to improve drug load and pharmacokinetics, allowing for a sustained release and enhanced accumulation at target sites [50,51]. Controlled drug release is a defining feature of advanced DDS. Systems that are designed with stimuli-responsive mechanisms release their payloads in response to endogenous triggers such as pH gradients, redox potential, enzymes, or ROS. In tumor microenvironments, for example, there are elevated ROS levels and an acidic pH compared to normal tissues, which give unique triggers for site-specific drug activation [52,53]. pH-responsive systems use these acidic microenvironments to trigger some structural changes in nanocarriers, such as swelling, dissociation, or cleavage of pH-labile bonds, which results in the localized drug release at tumor tissues or within acidic organelles such as endosomes and lysosomes [54]. These systems help prevent the premature leakage of drugs during circulation, which improves therapeutic effectiveness while limiting the exposure of healthy tissues. ROS-sensitive DDS, on the other hand, use the high oxidative stress in diseased tissues to trigger drug release. ROS-responsive polymer carriers contain motifs that are stable under physiological conditions but degrade in the presence of an increased number of ROS, which allows for an on-demand release of therapeutics [55]. Additionally, dual responsive systems that combine both ROS and pH sensitivity show synergistic release kinetics that are adapted to complex microenvironments, improving anticancer efficacy and minimizing off-target effects [56]. The rational design of nanomaterial-based drug delivery systems integrates advanced drug encapsulation strategies with stimuli-responsive release mechanisms to provide precise control over drug biodistribution and therapeutic action.

### 3.2. Delivery of Antioxidants and Redox-Modulating Therapeutics

The delivery of antioxidants and redox-modulating therapeutics has been transformed by nanomaterial-based DDS as they address fundamental limitations that are inherent to conventional antioxidant agents, such as unstable pharmacokinetics, rapid biological degradation, and the lack of tissue specificity. Nanomaterial antioxidant activity at the mechanistic level is driven by redox reactions that are surface-mediated. These reactions involve direct electron transfer and catalytic cycling. In order to neutralize reactive oxygen species, many nanoparticles act as electron acceptors or donors. Surface defect sites such as oxygen vacancies and undercoordinated atoms that serve as centers are key factors that control the efficiency. Recent studies that have been conducted on transition-metal-doped nanomaterials and engineered heterostructures show that redox and catalytic performance can have significant improvements through the regulation of the electronic structure and interfacial charge dynamics. When transition metals are incorporated, defect states are introduced and the band structure is modified. This supports electron transfer and further increases the density of catalytically active sites, such as oxygen vacancies. Similar to this is the formation of the heterostructure which generates built-in electric fields at the material interface. It will also promote more efficient charge separation and directional electron migration while keeping recombination well under control. All of these effects together improve catalytic redox cycling and all activities regarding ROS [57]. Continuous ROS scavenging through regenerative redox cycling is further supported by reversible valence transitions (Ce^3+^/Ce^4+^) in redox active systems such as cerium oxide. Additionally, the electron transfer kinetics can be governed by the local electronic structure and surface charge. This will be helpful in determining whether nanomaterials exhibit behavior that is antioxidant or pro-oxidant under certain biological conditions. Table 2 shows the traditional small molecule antioxidants, such as ascorbic acid or polyphenols, have often shown lower cellular uptake and rapid systemic clearance, which results in their clinical utility being restricted in oxidative stress-mediated diseases. Nanocarrier systems such as polymeric nanoparticles, liposomes, solid lipid nanoparticles, and hybrid platforms provide a means to protect, stabilize, and control the release of these small molecules, which improves therapeutic efficiency and bioavailability [58]. Small molecule antioxidants that are encapsulated within nanostructures have benefits such as increased protection against premature degradation and controlled release profiles that can extend systemic circulation and reduce the frequency of doses. These nanoscale systems can be engineered to have surface modifications that allow for better cell membrane penetration and target the tissue with a high level of oxidative stress [59]. For example, factors like stability and sustained activity have been improved by nanoencapsulation strategies and have given strong therapeutic results in preclinical models of oxidative stress [58]. Apart from small molecules, nanocarrier technology has also enhanced the delivery of protein-based antioxidants such as superoxide dismutase (SOD), catalase, and other endogenous enzymes. The encapsulation of enzymes within lipid–polymer hybrid nanoparticles or mesoporous silica systems has been shown to significantly improve enzyme stability and also protects against proteolytic degradation, which supports targeted delivery to the sites of inflammation or injury [60,61]. Such nanoformulations have shown increased retention in biological compartments and improved catalytic activity compared to free enzymes, thereby highlighting their potential for treating certain conditions such as inflammatory bowel disease and neurological oxidative damage [60]. Redox imbalances, for example, in tumor microenvironments with high glutathione levels, are used by stimuli-responsive nanocarriers to allow for the triggered release of therapeutic payloads and further precision in delivery [62]. These designs do not just facilitate the spatiotemporal control of antioxidant and redox-active drugs but also support the synergistic modulation of oxidative stress pathways in conjunction with conventional therapies. Importantly, the main factors that are driving the clinical interest in nanomaterial carriers are improved stability and bioavailability. By way of protecting labile therapeutics from degradation and supporting transport across biological barriers such as in mucus or in the blood–brain barrier (BBB), nanocarrier systems are able to achieve higher localized concentrations at target sites with a reduction in systemic exposure [59,60]. To ensure reproducibility and statistical reliability during the antioxidant activity evaluation, independent experimental replicates must be used. Assays such as 1,1-diphenyl-2-picrylhydrazyl (DPPH), 2,2′-azino-bis (3-ethylbenzothiazoline-6-sulfonic acid) (ABTS), or ROS scavenging measurements in most nanomaterial-based antioxidant studies are performed in triplicates or greater. The results are usually reported as mean ± standard deviation. Replicate measurements will reduce the variability of the experiment while also allowing for a better comparison between the different formulations. Whenever applicable, statistical analyses will also further support the validity of the differences that can be observed in the performance of antioxidants. Through the continued optimization of factors such as nanoparticle composition, surface chemistry, and targeting ligands, we can expect further improvements in the delivery of antioxidants and redox-modulating therapeutics in diverse disease contexts.

### 3.3. Pharmacokinetics and Biodistribution in Nanomaterial-Based Drug Delivery Systems

**Figure 4:**
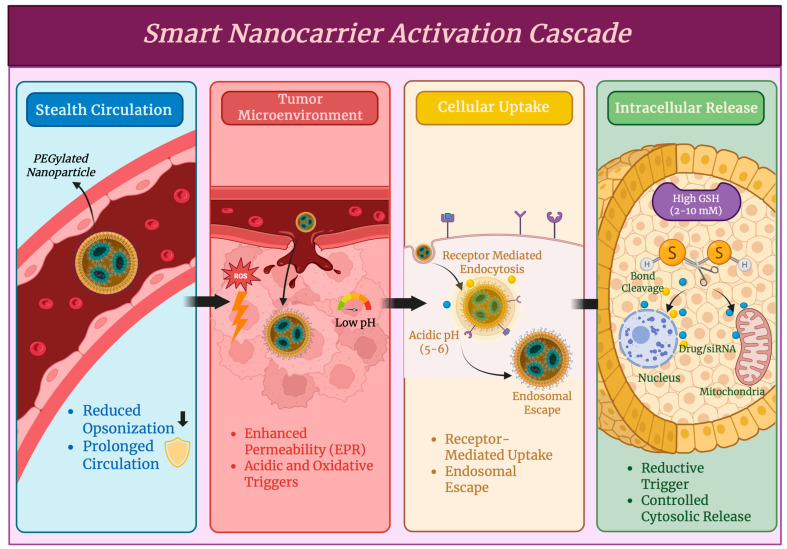
Schematic illustration of the sequential activation of a stimuli-responsive nanocarrier. After systemic administration, PEGylated nanoparticles avoid opsonization and prolong circulation. The accumulation at disease sites is due to improved permeability and retention (EPR), where the acidic pH and high reactive oxygen species (ROS) initiate structural destabilization. The following receptor-mediated endocytosis will allow for cellular internalization, followed by endosomal escape. High levels of intracellular glutathione (GSH) concentrations will trigger the cleavage of disulfide bonds, resulting in the controlled release of therapeutic payloads such as drugs or siRNA. This design allows for spatially and temporally regulated delivery while keeping off-target toxicity to a minimum. Created in BioRender. Roshan, D. (2026) https://BioRender.com/oj6a3ui, accessed on 21 March 2026.

In vivo pharmacokinetics and biodistribution profiles are important factors that determine the success of a given nanomaterial-based system, as they determine the therapeutic efficacy, dosing schedules, safety, and off-target toxicity. Nanocarriers can alter the traditional absorption, distribution, metabolism, and elimination (ADME) of drugs, as they are able to modify how the payload and carrier circulate and localize within tissues compared to small molecules [63]. The nanocarrier’s physicochemical properties, such as size, surface charge, surface chemistry, and composition, play a huge role in circulation time and tissue accumulation. Nanoparticles that are in the size range of 20–200 nm can often achieve longer systemic circulation as they avoid rapid renal filtration since they are larger than 10 nm, and excessive mononuclear phagocyte system (MPS) uptake since they are smaller than 200 nm, which allows for effective tissue accumulation and improved drug exposure at target sites [64]. Opsonization by serum proteins can be reduced by surface modification techniques such as polyethylene glycol conjugation (PEGylation) or zwitterionic coatings, as shown in Figure 4, and minimizes recognition by phagocytic cells and extends circulation half-life [65,66]. Blood residence has been prolonged, and tumor accumulation has been improved through passive mechanisms such as the enhanced permeability and retention (EPR) effect in tumor vasculature. Apart from passive targeting, active targeting through surface ligands, such as antibodies and peptides, is known to direct carriers to specific cellular receptors, leading to an increased local drug concentration and improved therapeutic index while also reducing distribution to healthy tissues [67]. However, effective tissue accumulation must balance carrier stability, controlled release, and target specificity to avoid issues such as premature clearance and nonspecific uptake [63]. The clearance and metabolism of nanomaterials is very different from conventional drug clearance. Nanoparticles are primarily removed by the MPS, particularly in the liver and spleen, and may also undergo size-dependent renal elimination if they are below the glomerular filtration threshold [64]. Designing biodegradable or renally clearable carriers is a major design objective, as the duration of systemic exposure and the potential for chronic toxicity are heavily influenced by organ clearance [63]. Biodistribution studies provide key PK data for translation by often using advanced imaging, radiolabeling, or mass spectrometry methods to measure organ-specific accumulation and elimination pathways [68]. The understanding and optimization of these pharmacokinetic and biodistribution parameters will be key in the advancement of nanomedicine directly to the bedside, improvements in the precision of drug delivery, and outcomes across oncologic, inflammatory, and genetic disease contexts [63].

**Table 2:** Summary of functional engineering strategies and therapeutic benefits of nanomaterial-based drug delivery systems.

DDS Functional Feature	Nanomaterial Strategy	Mechanistic Advantage	Example Therapeutic Application	Main Translational Benefit
Targeting modality (passive vs. active targeting) [69]	Ligand-modified liposomes, antibody-functionalized polymeric nanoparticles	Receptor-mediated endocytosis improves cellular specificity	Human epidermal growth factor 2 (HER2)-targeted breast cancer therapy	Reduced off-target toxicity and improved tumor uptake
Endosomal escape enhancement [70]	Ionizable lipid nanoparticles and proton sponge polymer systems	Disrupts endosomal membrane allowing for cytosolic payload release	mRNA and siRNA therapeutics	Increased nucleic acid delivery efficiency
Biological barrier penetration [71]	Surface-modified nanoparticles (PEGylation, cell-penetrating peptides)	Reduces opsonization and improves tissue diffusion	Blood–brain barrier delivery of neuroprotective drugs	Extended circulation and CNS drug delivery
Multidrug co-delivery systems [34]	Lipid–polymer hybrid nanoparticles or mesoporous silica nanocarriers	Allows for the synchronized delivery of multiple therapeutic agents	Combination chemotherapy and chemo-sensitizer delivery	Overcomes drug resistance and improves therapeutic synergy
Stimuli-responsive release (tumor microenvironment) [72]	ROS-responsive polymer carriers and pH-sensitive micelles	Controlled degradation that is triggered by pathological conditions	Tumor-selective chemotherapy	Spatially controlled drug activation
Immune system modulation and stealth delivery [73]	Cell membrane-coated nanoparticles (erythrocyte, cancer cell, or macrophage membranes)	Mimics natural biological surfaces to evade immune clearance	Inflammatory disease therapy and cancer targeting	Prolonged circulation and improved biodistribution
Enzyme/protein therapeutic stabilization [74]	Polymeric nanocapsules and silica-based enzyme carriers	Protects proteins from denaturation and proteolysis	Delivery of antioxidant enzymes such as SOD, catalase	Improved enzymatic activity and therapeutic half-life
Controlled pharmacokinetic tuning [75]	Biodegradable polymer nanoparticles (PLGA, polycaprolactone (PCL) systems)	Tunable degradation rate controls the sustained drug release	Long-acting injectable formulations	Reduced dosing frequency and improved patient compliance
Theranostic integration [76]	Metal–organic frameworks, gold or iron-oxide hybrid nanoparticles	Combines imaging and therapy into a single platform	Image-guided cancer therapy	Enables real-time treatment monitoring

## 4. Targeted Drug Delivery Strategies

### 4.1. Passive and Active Targeting

**Figure 5:**
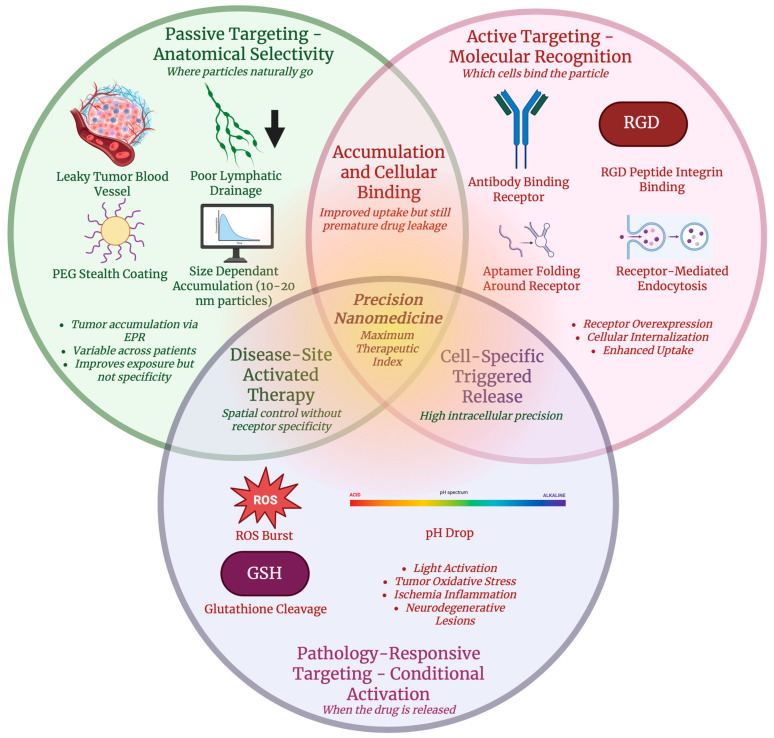
Venn diagram showing how modern nanocarriers have achieved therapeutic selectivity through the integration of three complementary mechanisms. Firstly, passive targeting through improved permeability and retention (EPR)-mediated accumulation in diseased tissues. Secondly, active targeting through ligand-receptor interactions that develop cellular binding and internalization. Lastly, pathology-responsive targeting, which is triggered by disease-associated microenvironmental signals such as ROS, acidic pH, or redox gradients. The overlapping regions represent higher levels of specificity, while the central intersection stands for precision nanomedicine, where spatial accumulation, cellular recognition, and conditional release all combine together to maximize the therapeutic efficiency and minimize off-target toxicity across cancer, ischemia, and neurodegenerative conditions. Created in BioRender. Roshan, D. (2026) https://BioRender.com/bb2sv5h, accessed on 21 March 2026.

Targeted drug delivery methods aim to accumulate therapeutic agents at disease sites while keeping systemic toxicity to a minimum. Biological relevance and translational applicability were key parameters for the selection of the targeting strategies discussed in this section. More focus was put on mechanisms that allowed for selective accumulation, receptor-mediated uptake, and microenvironment responsive activation. Passive targeting and active targeting are two paradigms that are foundational in nanomedicine and in the research of oncological drug delivery, which is further explained in Table 3 along with other targeted drug delivery methods [77]. Passive targeting utilizes the pathophysiological differences between diseased and healthy tissues. Abnormal vasculature with larger endothelial gaps, along with decreased lymphatic drainage in solid tumors, allows for macromolecules and nanoparticles typically sized between 10 and 200 nm to extravasate and preferentially accumulate in tumor interstitium, which is called the enhanced permeability and retention (EPR) effect [78]. The EPR effect has formed the basis for many nanocarrier-based therapeutics, including PEGylated liposomes such as Doxil^®^, that have shown improved tumor accumulation in comparison with free drugs through this mechanism [79]. However, an increasing number of limitations are observed with the EPR effect. Due to the differences in vascular density, interstitial fluid pressure, and stromal barriers, the intensity and uniformity of EPR-mediated accumulation vary significantly between tumor types, among patients, and even within different regions of the same tumor [80]. Moreover, since the EPR effect is more often pronounced in small animal models than in human tumors, it is difficult to translate many of these nanomedicines into successful clinical products [79]. To overcome these intrinsic physiological barriers, methods such as vascular modulation and imaging-guided delivery are being tested to improve or complement existing EPR [81]. Active targeting, on the other hand, builds on passive accumulation by functionalizing the surface of nanocarriers with molecular ligands that can bind specifically to receptors or antigens that are overexpressed on target cells or in the tumor microenvironment [82]. Active targeting attempts to increase selective uptake, and intracellular delivery of therapeutic payloads to increase overall safety and effectiveness by binding to specific receptors or molecular targets on cells [83]. Antibodies, peptides, and aptamers are some common targeting ligands, with each of them having their own benefits and limitations [84]. Antibodies and antibody fragments provide a high level of specificity and affinity for cell-surface markers such as epidermal growth factor receptor (EGFR) and HER2, and are often used in antibody-drug conjugates (ADCs) to precisely deliver cytotoxic agents to cancer cells [85]. Despite their benefits, their large size is a restriction and can limit the level of tissue penetration and also raises questions regarding immunogenicity [86]. Peptides such as cyclic arginylglycylaspartic acid (RGD) peptides that target integrins on tumor vasculature are smaller, easier to synthesize, and can penetrate tissues more readily, although it is often with lower affinities than antibodies [87]. Aptamers are short, single-stranded oligonucleotides that can fold into three-dimensional structures that bind to targets with high selectivity. Versatile conjugation to nanoparticles and drugs is possible due to their small size and chemical manufacturability, with recent reviews stating their potential as smart ligands in targeted cancer therapy [88]. Active targeting can work together with passive targeting, which is depicted in Figure 5, where nanocarriers first accumulate at tumor sites through EPR, then engage specific cellular receptors through ligand binding to improve cellular internalization and retention [89].

### 4.2. Targeting Oxidative Stress-Associated Microenvironments

Oxidative stress is defined as the imbalance between reactive oxygen species generation and antioxidant defenses and is also considered a hallmark of various pathological microenvironments, such as tumors, inflamed or ischemic tissues, and neurodegenerative disease sites. When these redox abnormalities are exploited by drug delivery strategies, it can allow for selective activation or the accumulation of therapeutics at disease sites while keeping systemic toxicity to a minimum [90,91]. The tumor microenvironment (TME) shows increased ROS levels and redox homeostasis that is altered compared to normal tissues, which create an opportunity for ROS-responsive drug carriers to perform site-specific delivery and release. The ROS-responsive liposomal systems are engineered in such a way that higher oxidative levels in the tumor trigger structural changes and controlled drug release, thereby improving therapeutic selectivity and efficiency [92]. Targeted redox-manipulating nanocarriers can selectively intensify the oxidative stress within tumor cells either by photodynamic agents generating ROS or by depleting antioxidant defenses such as glutathione, which pushes for tumor cell death while sparing normal healthy tissue [93]. Transferrin receptor-targeted nanosystems is one of the recent advancements that generates ROS under near-infrared light to break ROS-responsive linkages and increase payload release in tumors, which is helpful for phototherapy and immunogenic tumor cell killing [94]. Inflammation and ischemia further intensify oxidative stress by producing excessive ROS, which makes ROS-sensitive delivery systems particularly more attractive. Nanomedicines that are designed to target inflammatory cells in the ischemic myocardium have the ability to deliver antioxidants, for example quercetin, neutralize ROS, and promote a reparative macrophage phenotype that will improve cardiac repair [95]. In cerebral ischemia–reperfusion injury, ROS-targeted nanomedicines that can penetrate the ischemic penumbra and deliver antioxidant agents have shown potential in diminishing oxidative neuronal damage and reperfusion injury [96]. Similarly, ROS-responsive systems that were designed for brain ischemia can cross the blood–brain barrier and selectively search for ROS to regulate the post-ischemic inflammation and also maintain neuronal function [97]. Reviews of antioxidant nanotherapies have stated that there was limited efficacy due to poor targeting in conventional antioxidant drugs, which is an issue that ROS-responsive carriers can control in inflammatory disease contexts [98]. Neurodegenerative diseases such as Alzheimer’s, Parkinson’s, and other related disorders are closely linked to chronic oxidative stress, which can cause protein aggregation, mitochondrial dysfunction, and neuronal death [90]. Nanotechnology-based, stimuli-responsive systems, such as the ROS-sensitive carriers, have improved the delivery of neuroprotective agents across the blood–brain barrier and released them when signaled by oxidative cues at disease sites [99]. Such platforms provide improved specificity and reduce systemic side effects that are often seen with conventional therapies [100]. Many pathophysiological features, such as oxidative damage, inflammation, and protein aggregation, can be simultaneously addressed by ROS-responsive nanocarriers that are designed to detect oxidative microenvironments in neurodegenerative lesions and thus hold significant therapeutic potential [101].

**Table 3:** Translational performance of targeted drug delivery strategies and their comparative design principles.

Targeting Strategy	Targeting Ligand or Trigger	Biological Target or Microenvironment	Example of Delivery Vehicle	Quantitative Performance Metric	Clinical/Translational Status
Passive Targeting (EPR-based accumulation) [7]	Size optimization, PEG stealth coating	Leaky tumor vasculature, impaired lymphatic drainage	PEGylated liposomes, for example Doxil^®^	Tumor to plasma drug concentration ratio, circulation half-life	Clinically approved nanomedicines
Antibody-Mediated Active Targeting [102]	Monoclonal antibodies such as anti-HER2, anti-EGFR	Overexpressed tumor cell surface receptors	Antibody-drug conjugates or antibody-functionalized nanoparticles	Binding affinity (Kd), receptor internalization rate	Multiple FDA-approved ADCs
Peptide-Based Targeting [103]	RGD peptides, tumor-homing peptides	Integrins and angiogenic endothelial markers	Polymeric nanoparticles, liposomes	Cellular uptake efficiency, endothelial adhesion rate	Several preclinical and early clinical trials
Aptamer-Guided Targeting [104]	Nucleic acid aptamers for example AS1411 nucleolin aptamer	Cancer cell membrane proteins or tumor stromal markers	Aptamer-decorated lipid or polymeric nanocarriers	Ligand specificity index, target-to-non-target uptake ratio	Emerging clinical-stage ligand platforms
Redox/ROS-Responsive Targeting [105]	ROS-cleavable linkers, thioketal polymers	High oxidative stress tumor or inflammatory microenvironments	ROS-sensitive polymer micelles or liposomes	Triggered release percentage under ROS conditions	Rapidly developing preclinical development
pH-Responsive Targeting [106]	Acid-cleavable linkers, protonatable polymers	Acidic tumor interstitium or endosomal compartments	pH-sensitive polymeric nanoparticles or micelles	Drug release rate at pH 5–6 vs. physiological pH	Preclinical and translational research stage
Biomimetic/Cell Membrane Targeting [107]	Cancer-cell, macrophage, or erythrocyte membrane coatings	Immune evasion and homologous tumor recognition	Cell membrane-coated nanoparticles	Circulation time extension, immune clearance reduction	Early translational and experimental stage

## 5. Nanomaterials for Gene Delivery and Genetic Therapeutics

### 5.1. Challenges in Gene Delivery and Lipid, Polymeric, and Inorganic Nanocarriers

One of the most difficult challenges in genetic therapeutics is effective gene delivery, which is mainly due to the biological barriers that limit the stability, cellular uptake, and intracellular trafficking of nucleic acids. Nanocarrier systems that have been discussed here, were prioritized based on their efficiency in overcoming any key intracellular barriers. These include their compatibility with clinically relevant nucleic acid therapeutics, as well as endosomal escape and cytosolic release. Naked DNA, mRNA, and siRNA are very susceptible to enzymatic degradation in biological fluids and show poor permeability across cellular membranes, which causes rapid clearance and therapeutic engagement that is often not enough. The stability of genetic cargo in systemic circulation and protection from nuclease degradation is very important, as therapeutic nucleic acids can be quickly degraded before they reach the target cells without vector protection [108]. For cellular uptake to be efficient, it needs nanocarrier surfaces to interact with negatively charged cell membranes, which requires modified surface chemistries and targeting ligands [109]. A critical hurdle after internalization through endocytosis is endosomal escape, which is the movement of genetic cargo from the vesicles into the cytosol before lysosomal degradation. Endosomal entrapment will reduce therapeutic efficiency, which has pushed for extensive research into pH-responsive nanomaterials that can facilitate endosomal membrane disruption or fusion [110]. Currently, not even advanced lipid nanoparticles (LNPs) can achieve a high percentage of endosomal escape success, which further highlights the need for designs that escape endosomes effectively and safely [111]. Today, lipid-based nanocarriers, including LNPs and liposomes, are the most clinically advanced non-viral delivery platforms. Ionizable lipids that are neutral in physiological pH but become positively charged in acidic endosomes, have the ability to interact with endosomal lipids, help with membrane fusion, and stimulate the release of nucleic acid [112]. As LNPs had shown scalability and high delivery efficacy, particularly in siRNA and mRNA applications, they are considered central to the success of modern nucleic acid therapeutics [108]. Gene payloads and their nanomaterial platforms are discussed in Table 4. However, lipid systems still have challenges surrounding limited endosomal escape efficiency and their stability during storage, which calls for further development [111]. Polymeric nanocarriers have tunable physicochemical properties and often show better stability and prolonged circulation levels in comparison to lipids. Cationic polymers such as polyethylenimine (PEI) and poly (β-amino esters) can condense nucleic acids into polyplexes and allow for cellular uptake, although cytotoxicity and reproducibility remain as obstacles. Up-and-coming polymeric micellar structures and block copolymers have the potential to improve endosomal disruption through proton sponge effects or membrane destabilization, which will provide better safety and efficiency [113]. Inorganic nanocarriers such as gold, silica, and magnetic nanoparticles are other platforms with unique imaging and functionalization features. While functioning as theranostic agents, these materials can also deliver nucleic acids; however, there are concerns surrounding their biocompatibility and long-term toxicity [114]. They have rigid cores that provide structural stability and surface modifications with polymers or peptides that are used to improve uptake and endosomal escape. Despite several advancements in all classes of nanocarriers, challenges remain, mainly around scalable manufacturing, predictable biodistribution, targeted delivery, and having a high cytosolic release of genetic cargo that is required for strong therapeutic outcomes [109].

### 5.2. Genetic Therapeutics and Redox-Responsive Gene Delivery

**Figure 6:**
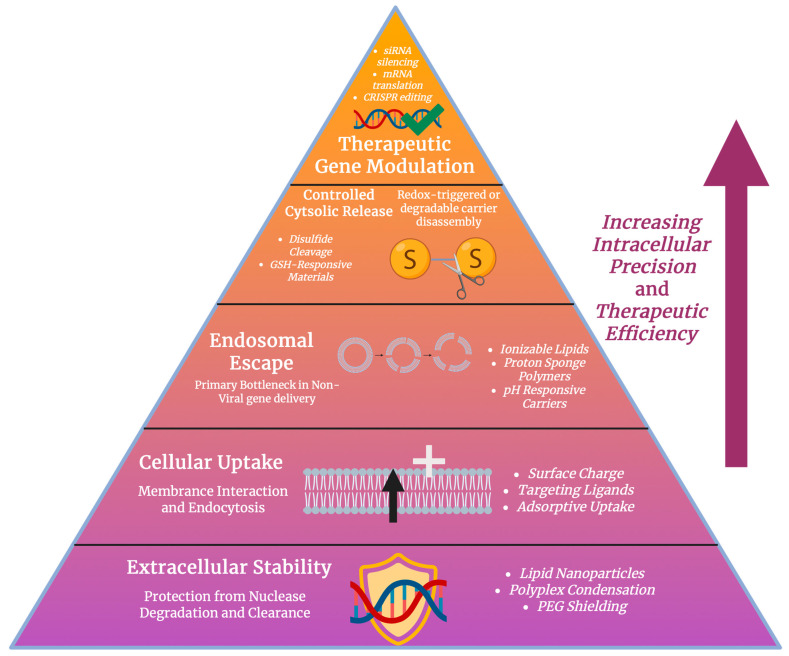
The requirements for achieving functional nucleic acid therapeutics are shown in the pyramid diagram. After entering systemic circulation, nanocarriers must be able to protect genetic cargo from extracellular degradation, allow for cellular uptake through membrane interactions, overcome endosomal entrapment, which is the biggest challenge in non-viral delivery, go through stimuli-responsive intracellular disassembly to release cargo into the cytosol, and ultimately provide therapeutic gene modulation, including siRNA silencing, mRNA translation, or CRISPR-mediated editing. Created in BioRender. Roshan, D. (2026) https://BioRender.com/fjxez6p, accessed on 21 March 2026.

Nanomaterials have transformed gene delivery and genetic therapeutics by allowing for the efficient and targeted transport of nucleic acid cargos such as siRNA, mRNA, and clustered regularly interspaced short palindromic repeats (CRISPR/Cas) components. Nanocarriers overcome issues usually faced by regular delivery methods, such as poor stability, limited cellular uptake, and the rapid degradation of nucleic acids, through their physicochemical properties that improve circulation, targeting, and intracellular release [115]. siRNA mediates specific gene silencing through RNA interference but also requires effective carriers to protect it from nucleases and allow for cellular uptake. Studies have been widely conducted on nanocarriers such as lipid nanoparticles, polymers, and inorganic nanostructures for this purpose [116]. Lipid and polymeric core–shell nanoparticles provide an improvement in stability and endosomal escape, which enhances the gene knockdown efficacy [117]. Redox-responsive polymers and nanomaterials that respond to high intracellular glutathione concentrations allow for the controlled release of siRNA within target cells, thus improving silencing potency [118]. mRNA therapeutics require carriers that can protect the negatively charged, large molecules and deliver them to the cytosol for translation, as seen in Figure 6. Lipid nanoparticles are now considered as the predominant platform for mRNA vaccines and therapeutics, with tunable lipid compositions supporting organ targeting, cellular uptake, and the controlled release of cargo [119]. The therapeutic index for both vaccination and protein replacement strategies can be increased by programmable lipid nanoparticles that are engineered to improve organ tropism and intracellular trafficking [120]. CRISPR/Cas systems present huge therapeutic potential when used in precision genome editing, although there are challenges surrounding the delivery of CRISPR components such as Cas protein or mRNA and guide RNA to specific tissues. To encapsulate and deliver these components safely and efficiently, nanomaterials such as polymeric, lipid, and inorganic carriers have been adapted [121]. Redox-specific nanomaterials use the differences between extracellular and intracellular redox potential, particularly the high glutathione levels inside cells, to trigger the release of cargo [122]. Nanocarriers containing disulfide and diselenide bonds are stable in circulation but disintegrate quickly when inside the reducing intracellular environment, which supports the on-demand release of siRNA, mRNA, or CRISPR reagents. To exploit this glutathione gradient between extracellular and cytosolic compartments, mesoporous silica carriers, polymersomes, and polymeric micelles have been designed with redox-cleavable linkages that help with cytoplasmic delivery [123]. Nanomaterials can also interact with cellular antioxidant systems. Redox-manipulating carriers can reduce the levels of intracellular glutathione to sensitize cancer cells or regulate oxidative stress pathways, which will improve therapeutic efficacy when co-delivering genetic cargo [62]. Antioxidant nanoparticles, including nanoceria, have displayed redox sensitivity that can be controlled for responsive release and for the therapeutic regulation of oxidative pathways [122]. Nanomaterials for gene delivery have provided versatile platforms for siRNA, mRNA, and CRISPR/Cas therapeutics, with redox-responsive systems having precise control over intracellular release, which is particularly important in diseases with aberrant antioxidant and redox pathways.

**Table 4:** Intracellular delivery mechanisms, translational readiness, and safety factors of nanomaterial platforms for genetic therapeutics.

Type of Gene Payload	Nanomaterial Platform	Primary Intracellular Release Mechanism	Main Efficient Design Feature	Major Safety or Translational Limitation	Representative Therapeutic Application
siRNA [124]	Lipid Nanoparticles (LNPs)	Ionizable lipid-mediated endosomal membrane fusion	pH-responsive lipid protonation enhances cytosolic release	Limited escape efficiency, inflammatory responses	Gene silencing in liver metabolic disorders
siRNA [125]	Redox-responsive polymeric nanoparticles	Glutathione-triggered disulfide cleavage	Intracellular redox gradient exploitation for controlled siRNA release	Batch reproducibility and polymer toxicity concerns	Tumor gene knockdown therapy
mRNA [126]	Programmable lipid nanoparticles	Endosomal destabilization and membrane fusion	Tunable lipid composition controlling organ tropism	Storage instability and temperature sensitivity	mRNA vaccination and protein replacement therapy
mRNA [127]	Polymer–lipid hybrid nanoparticles	Dual release via lipid fusion and polymer degradation	Structural stabilization improves circulation and cargo protection	Manufacturing scale complexity	Cancer immunotherapy and antigen expression
CRISPR/Cas RNP [128]	Polymeric nanocapsules or micelles	Proton sponge-induced osmotic swelling and vesicle rupture	Efficient condensation of Cas protein and sgRNA	Potential cytotoxicity from cationic polymer density	Precision genome editing in inherited diseases
CRISPR/Cas mRNA/sgRNA [129]	Lipid nanoparticles	Endosomal fusion releasing translation-ready mRNA	High transfection efficiency and transient expression safety	There is a risk of Off-target editing	Gene editing for transthyretin amyloidosis
Multi-gene payload delivery [130]	Mesoporous silica nanoparticles	Redox-cleavable gatekeepers controlling pore release	High loading capacity with simultaneous cargo delivery	Slow biodegradation and long-term tissue accumulation	Combination gene regulation and drug co-delivery
siRNA or CRISPR components [131]	Gold nanoparticle conjugates	Surface ligand cleavage or photothermal-triggered release	Precise surface functionalization and imaging integration	Long-term accumulation and clearance concerns	Theranostic gene delivery and photothermal therapy
Gene cargo with antioxidant modulation [132]	Nanoceria-integrated hybrid carriers	ROS/redox-regulated carrier degradation	Simultaneous oxidative stress modulation and gene release	Environment-dependent catalytic activity variability	Neurodegenerative and inflammatory gene therapy

## 6. Theranostic Nanoparticles for Integrated Therapy and Diagnosis

### 6.1. Design and Applications of Imaging-Guided Theranostic Nanocarriers

**Figure 7:**
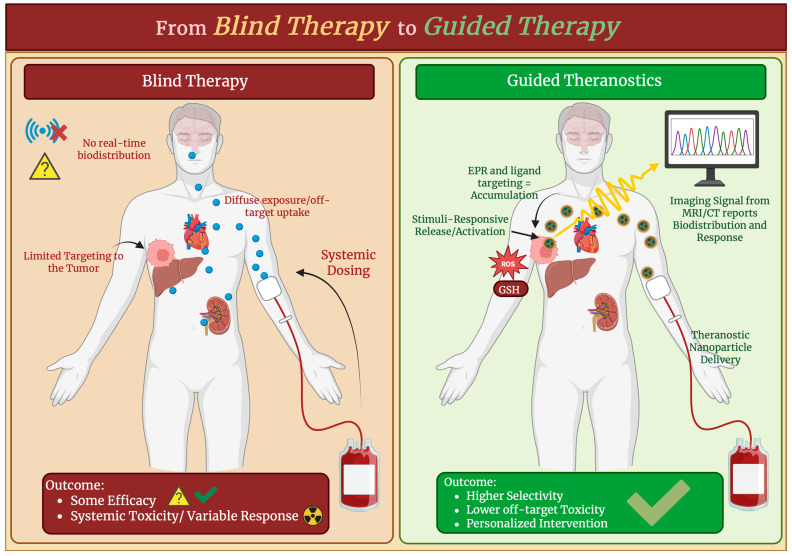
Conventional systemic drug administration only offers diffuse biodistribution, limited targeting, and a lack of real-time treatment monitoring, which leads to a variable efficacy and off-target toxicity. In comparison, theranostic nanoparticles co-deliver therapeutic and imaging components, accumulate at diseased tissue through passive and ligand-mediated targeting, and undergo microenvironment-triggered activation. The imaging signal allows for the real-time assessment of biodistribution and therapeutic response, making the adaptive adjustment of treatment strategy and closed-loop precision intervention possible. Created in BioRender. Roshan, D. (2026) https://BioRender.com/5w5c63j, accessed on 21 March 2026.

Theranostic nanoparticles are part of an advanced class of multifunctional nanomedicine that are designed to combine diagnostic imaging and therapeutic delivery in a single platform to give more precise and individualized treatment strategies. The selected theranostic systems here were chosen based on their ability to integrate imaging and therapy within a single platform. They should be able to do this while maintaining the biological relevance and functional responsiveness to disease-associated microenvironments such as oxidative stress. The various different types of theranostic platforms and their imaging modalities can be seen in Table 5. In order to support the real-time monitoring of drug delivery, biodistribution, and therapeutic efficacy, a high level of importance is placed on the integration of imaging agents such as MRI contrast moieties and fluorescence dyes with drugs, genes, photothermal elements, or other therapeutic payloads. Through this dual function, some limitations of conventional therapies can be addressed by providing spatial and temporal insights into the progression of treatment while also improving targeted therapy at disease sites such as tumors [133]. Many different design principles make up the concept and design of theranostic nanoparticles. One of which is that the nanoparticle must have a multimodal architecture that can carry and release therapeutic agents in response to specific biological cues such as pH levels and enzymes while also providing detectable imaging signals. Some examples include polymeric carriers, liposomes, metal and metal-oxide cores, and mesoporous silica frameworks that are functionalized with targeting ligands to utilize the EPR effect or active ligand-receptor interactions for tumor localization [134]. Another key design principle is the integration of imaging contrasts, including optical, magnetic, and nuclear, as it allows for the visualization of nanoparticles in vivo behavior, which is necessary to diagnose disease states and image-guided therapy [135]. As seen in Figure 7, these theranostic platforms are used by imaging-guided drug and gene delivery to direct and monitor therapeutic intervention. For example, when under image guidance, nanoplatforms that co-deliver chemotherapeutic agents and gene silencers have shown a higher anticancer efficacy with off-target effects that are minimal in preclinical models [136]. In another study, HER-2-targeted theranostic nanoparticles were loaded with cisplatin and had allowed for the non-invasive MRI tracking of drug delivery and therapeutic responses in heterogeneous ovarian cancer models [137]. Image-guided surgery and targeted drug delivery were demonstrated by nanotheranostic H-dots, which helped improve surgical precision and therapeutic outcomes [138]. Theranostic nanoparticles have also been engineered for gene therapy integration, where multifunctional platforms allow for the simultaneous delivery of DNA or siRNA with imaging tracers, which helps with the spatial control of gene modulation while tracking the delivery status through imaging modalities [134].

### 6.2. Redox-Responsive Theranostic Nanoparticles for Disease-Targeted Imaging and Treatment

The unique biochemical features of different pathological environments, such as elevated ROS in tumors or oxidative stress in neurodegeneration, allow for the selective activation or modulation of nanoparticle function, which improves specificity and reduces off-target effects. The tumor microenvironment in cancer is characterized by its high levels of ROS and glutathione. This oxidative imbalance is exploited by specifically designed redox-responsive nanocarriers that trigger the release of the payload and simultaneous imaging signal activation. These systems often combine ROS-sensitive linkages or catalysts with imaging agents to improve contrast in magnetic resonance or optical imaging while also increasing cytotoxic ROS levels or reducing antioxidant defenses to promote the death of tumor cells [139]. An example use of this strategy had achieved integrated imaging and therapy in preclinical cancer models through copper ferrite nanospheres that generate ROS from photoenhanced Fenton reactions and provided high relaxivity for MRI [140]. TME signals are also exploited to trigger release and immune activation against colon tumors by ROS-sensitive mesoporous silica nanoparticles that co-deliver immunogenic components [141]. In neurological disorders such as Alzheimer’s, chronic oxidative stress is a defining pathological factor. Recently, there has been an up-and-coming field of antioxidant nanomedicines that are designed to scavenge ROS while allowing for diagnostic imaging. For example, ROS-scavenging nanoparticles with intrinsic antioxidant activity or loaded antioxidants have shown potential in reducing oxidative damage, regulating the pathology of amyloids, and behaving as contrast agents for neuroimaging [142]. Nanocarriers that are capable of crossing the blood–brain barrier while simultaneously delivering therapeutic and diagnostic functions are key highlights in the field of theranostic nanotechnology in neurodegenerative diseases [143]. Overall, a continued development of redox-sensitive chemistries, biocompatibility, and imaging modalities will be essential for its clinical translation (Table 5).

**Table 5:** Various theranostic nanoplatforms and their imaging modalities, signal activating strategies, and translational advantages.

Theranostic Nanoplatform Type	Integrated Imaging Modality	Therapeutic Mechanism	Signal Activation Strategy	Disease Application Model	Translational or Clinical Advantage
Gold nanorods/nanoshells [144]	Photoacoustic imaging, CT, optical imaging	Photothermal therapy (PTT), drug delivery	Localized surface plasmon resonance enhancing photothermal conversion and imaging contrast	Solid tumors and metastatic cancers	Allows for real-time optical/thermal tumor ablation monitoring
Iron oxide nanoparticle hybrids [145]	Magnetic resonance imaging	Magnetic hyperthermia, drug or gene delivery	Superparamagnetic relaxation signal enhancement and heat generation under alternating magnetic field	Brain tumors, prostate cancer	Clinically translatable MRI compatibility and magnetic targeting
Mesoporous silica theranostic nanoparticles [146]	Fluorescence imaging, MRI, PET (when radiolabeled)	Controlled chemotherapy or immunotherapy release	Stimuli-responsive gatekeepers such as ROS, pH, enzyme-triggered pore opening	Colon cancer and inflammatory disease	High cargo loading and multiplex therapeutic delivery
Metal–organic framework (MOF) nanoplatforms [147]	MRI, fluorescence, radionuclide imaging	Photodynamic therapy (PDT), chemotherapy, enzyme-mimetic therapy	Porous catalytic ROS generation enhancing imaging and therapeutic cytotoxicity simultaneously	Hypoxic tumors and drug-resistant cancers	High tunability of composition and multifunctional payload integration
Upconversion nanoparticle-based theranostics [148]	Near-infrared fluorescence imaging	Photodynamic therapy and gene delivery	NIR-to-visible photon conversion allowing for deep tissue imaging and controlled drug activation	Deep-seated tumors and orthotopic cancer models	Allows deeper tissue penetration with low autofluorescence background
Copper or manganese-based Fenton catalytic nanoparticles [149]	MRI, photoacoustic imaging	Chemodynamic therapy (ROS-generating Fenton reactions)	Tumor microenvironment-activated ROS generation raises imaging signal and cytotoxicity	Aggressive or hypoxic tumors	Microenvironment-responsive activation improves specificity
Lipid–polymer hybrid theranostic nanoparticles [150]	Fluorescence imaging, MRI or radionuclide labeling	Co-delivery of chemotherapy and nucleic acids (siRNA/mRNA)	Stimuli-responsive polymer degradation combined with lipid fusion-mediated cytosolic release	Multidrug-resistant cancers	Supports the combination of gene-drug therapy with imaging-guided dosing
Nanoceria or antioxidant redox-responsive nanoparticles [151]	MRI or optical redox imaging	ROS scavenging, neuroprotective or anti-inflammatory therapy	Redox cycling catalytic activity allowing self-regenerating antioxidant imaging probes	Neurodegenerative diseases and ischemic injury	Simultaneous disease biomarker detection and oxidative stress therapy

## 7. Limitations, Safety, and Future Perspectives Discussion

### 7.1. Safety and Toxicological Considerations

The unique physicochemical properties of nanomaterials in therapeutic delivery pose some distinct safety concerns. A major issue in nanomedicine is nanomaterial-induced oxidative stress, where cellular damage, mitochondrial dysfunction, and lipid peroxidation are caused by ROS generation. This is explained further along with other limitations in Table 6. Studies have shown that metal and metal-oxide nanoparticles can significantly increase oxidative markers, disrupt protein function, and induce apoptosis, which requires the need of a careful assessment of redox balance in design strategies [152]. Oxidative stress also causes further implications in neurological and vascular toxicity, where an elevated ROS will adversely alter cellular pathways, which pushes for the need for antioxidant migration strategies during development [153]. Immunogenicity and long-term effects are also very important, as engineered nanocarriers can be recognized as foreign bodies by immune systems, causing immunostimulation, immunosuppression, or other hypersensitivity reactions. Immune activation will reduce efficacy and produce side effects such as complement activation or cytokine release, highlighting the need for nano-immune interaction profiling [154]. The long-term safety of nanomaterials in therapeutic delivery is not fully understood either. The chronic exposure of degradable nanomaterials can lead to tissue accumulation, alterations in the microbiome, and organ perturbations, and the byproducts of biodegradable carriers affect immunogenicity and barrier integrity unpredictably [155]. To address these complicated risks, serious regulatory considerations and standardized toxicological assays must be implemented [156]. Mechanistic toxicology and advanced nanodesign should be integrated into future designs, using strategies like predictive models and surface modifications to reduce oxidative and immune related toxicities [157].

### 7.2. Translational and Regulatory Barriers in Nanotherapeutics

Significant translational and regulatory challenges block the clinical translation of nanomaterials for therapeutic delivery, particularly by issues like manufacturing scalability and regulatory approval pathways. Nanomedicines often have complex physicochemical properties that are difficult to consistently reproduce at an industrial scale. Size, surface charge, and drug loading can vary batch-to-batch, affecting pharmacokinetics, biodistribution, and therapeutic outcomes, which makes the transition from laboratory synthesis to good manufacturing practice (GMP) production a hurdle [158]. On top of the general manufacturing challenges, batch-to-batch reproducibility and synthesis yield are critical issues that must be evaluated for nanomaterial translation. It is through independent synthesis batches that are under controlled conditions, the reproducibility is assessed. This ensures that there will be consistency in physicochemical characteristics such as the distribution of particle size, morphology, and surface charge [159]. The performance of nanomaterials can be significantly affected by even the smallest variations in synthesis parameters such as precursor concentration, reaction temperature, and mixing dynamics [160]. The reporting of synthesis yield is also an equally important factor. It shows how efficient and scalable it is for clinical production. This is also why it is important to implement Quality-by-Design (QbD) frameworks so that we can ensure batch consistency is reliable and the biological outcomes are reproducible [161]. The control of critical quality features and the reproducibility of manufacturing processes are crucial for adoption on the industrial level, yet they remain poorly standardized [162]. Regulatory frameworks are also using conventional drug approval pathways for nanomedicines, which is an insufficient way of addressing their individual nanoscale behaviors, immunogenicity, or biodistribution profiles [163]. Since there are no internationally set guidelines, assessments are made case-by-case by authorities such as the FDA and EMA, which block global development strategies [158]. There have been recommendations like Quality by Design (QbD) and risk-based approaches to ensure safety and efficacy, but they are not widely adopted [164]. Dual evaluations under different regulatory centers are required as regulatory pathways of nanomedicine must withstand products that are combination therapeutics and devices [165]. Additionally, appropriate analytical methods for nanocharacterization and predictive preclinical models are still developing and further add to approval timelines [158]. Structural characterization also plays a major factor and must be reported with greater standardization. This is especially important when it comes to crystalline inorganic and hybrid nanomaterials. Studies that utilize X-ray diffraction (XRD) to estimate the size of the crystallite must clearly discuss the Scherrer equation parameters [166]. These include the shape factor (K), X-ray wavelength (λ), full width at half maximum (β), Bragg angle (θ), and whether or not instrumental broadening correction was done [167]. Phase purity should also be assessed in comparison with the reference diffraction patterns. Any secondary reflections that could possibly indicate impurity phases, incomplete crystallization, or residual precursor-derived materials should also be kept in consideration. This kind of reporting is crucial as structural heterogeneity may affect important parameters such as redox behavior, colloidal stability, and batch reproducibility. All of these factors will ultimately decide the overall translational reliability [168]. Academia, industry, and regulators must come together and form strategic collaborations to address these concerns to make nanotherapeutic innovations accessible to patients [169].

### 7.3. Future Directions in Therapeutic Nanomedicine

**Figure 8:**
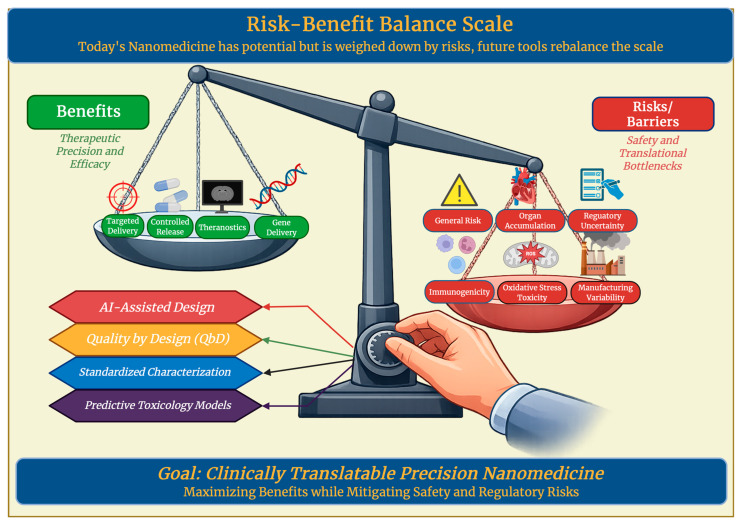
Nanocarrier platforms provide major benefits such as targeted delivery, controlled release, theranostic monitoring, gene cargo transport, and redox modulation, but are counterweighted by the safety risks that include oxidative stress, immunogenicity, and long-term accumulation, and translational hurdles like manufacturing variability and regulatory uncertainty. The developing solutions such as AI-assisted nanomaterial design, Quality-by-Design Manufacturing, standardized nanocharacterization, and predictive toxicology aim to rebalance this scale for clinically translatable precision nanomedicine. Created in BioRender. Roshan, D. (2026) https://BioRender.com/1tknc4h, accessed on 21 March 2026.

The future perspectives for nanomaterials in therapeutic delivery mainly surround personalized and precision nanomedicine, AI-assisted design, and upcoming antioxidant nanotherapies. In personalized nanomedicine, biological data from a specific patient is integrated with smart nanocarriers to provide therapy to an individual’s disease profile. Recent advances have shown programmable nanoparticles that utilize omics and imaging data for active targeting and controlled release, which is further described in Figure 8, showing a shift from conventional passive carriers to truly precision-guided platforms in oncology [170]. Tumor heterogeneity and microenvironmental barrier are both addressed by precision nanomedicine strategies that will improve immunotherapy and drug delivery with better outcomes [171]. Artificial intelligence (AI)-assisted nanomaterial designs optimize nanoparticle properties and predict the nano-bio interactions, which would benefit therapeutics in the future. Across synthesis, pharmacokinetics, and biodistribution predictions, machine learning models have been used, which significantly accelerated design cycles and supported adaptive carrier systems [172]. Reviews have also highlighted how deep learning and graph neural networks can reduce experimental workload and improve predictive accuracy in nanocarrier performance [173]. Moreover, intelligent nanoplatforms that were engineered with AI showed potential in synergizing diagnosis and treatment with multifunctional systems [174]. Given these advances, challenges remain in standardizing data frameworks and regulatory pathways that make AI-assisted nanomedicine difficult to implement [173]. The emerging antioxidant nanotherapies are particularly interesting as they target reactive oxygen species (ROS) and oxidative stress, which are key factors in inflammation and disease progression. Traditional antioxidants are affected by poor bioavailability and non-specific distribution, whereas antioxidants based on nanoparticles have given improved targeting, stability, and intrinsic ROS-scavenging capacity [98]. Nanozymes with catalase, SOD, and GPx-like activities are examples of rational designing being used to regulate oxidative pathways [175]. Moving forward, research should focus more on safety, controlled activation, and clinical translation so that we can realize the full therapeutic potential of antioxidant nanomedicines [176].

**Table 6:** Safety assessment and translational risk-mitigation strategies in nanotherapeutics.

Limitation Category	Assessment Model or Tool	Key Parameters Measured	Example Nanomaterial Context	Translational Relevance
Oxidative stress and redox imbalance [177]	High-content ROS imaging and mitochondrial stress assays	Intracellular ROS, ΔΨm, lipid peroxidation, Nuclear factor erythroid 2-related factor 2 (NRF2) activation	Metal/metal-oxide nanoparticles	Early hazard identification and redox profiling
Complement activation and Complement activation-related pseudoallergy (CARPA) [178]	Human whole-blood complement assays	C3a, C5a, SC5b-9 levels	Liposomes, lipid nanoparticles	Predict infusion reactions
Protein corona unpredictability [179]	Quantitative proteomics corona mapping	Corona composition, opsonin enrichment	PEGylated nanoparticles	Improves PK predictability
Long-term accumulation [180]	Radiolabel tracing and Inductively Coupled Plasma Mass Spectrometry(ICP-MS) biodistribution	Organ retention kinetics	Gold, silica nanoparticles	Chronic toxicity forecasting
Immunotoxicity [181]	Humanized mouse models	Cytokine release, adaptive immune activation	Polymeric carriers	Better human immune prediction
Blood–brain barrier safety assessment [182]	Microfluidic human BBB-on-chip	Transepithelial Electrical Resistance (TEER), permeability coefficient	Neuro-nanotherapeutics	Predict CNS toxicity
Manufacturing variability [183]	Quality-by-Design (QbD) frameworks	Critical Quality Attributes (CQA)	PLGA, lipid nanoparticle systems	Good Manufacturing Practice (GMP) reproducibility
Analytical nanocharacterization gaps [184]	Multi-angle dynamic light scattering, Nanoparticle Tracking Analysis (NTA), and Cryogenic Transmission Electron Microscopy (cryo-TEM) standardization	Size distribution, aggregation index	Hybrid nanoparticles	Batch consistency validation
Regulatory classification ambiguity [185]	Risk-based regulatory science model	Product categorization criteria	Theranostic nanoparticles	Harmonized approval pathway
AI-driven safety prediction [186]	Machine learning nano-quantitative structure–activity relationship (QSAR) modeling	Structure–toxicity relationships	Engineered nanomaterials	Preclinical risk reduction
Microbiome interaction [187]	16S rRNA sequencing after nanoparticle exposure	Gut flora diversity changes	Oral nanocarriers	Long-term systemic safety
Redox-adaptive design [177]	Catalytic antioxidant coatings	ROS modulation threshold	Nanoceria hybrids	Minimized oxidative cytotoxicity

## 8. Conclusions

Nanomaterials have proved themselves as transformative platforms in modern therapeutic delivery, as they now provide great control over drug, antioxidant, and genetic payload transport across complex biological barriers. Nanoscale systems have addressed some persistent limitations of conventional therapeutics, such as poor solubility, rapid degradation, limited bioavailability, and off-target toxicity, by intelligently engineering their size, surface chemistry, charge, and stimuli-responsiveness. These nanoparticles help with controlled release, improved pharmacokinetics, and better accumulation at disease sites through methods like passive and active targeting mechanisms. A particularly significant development is that of redox-responsive and ROS-sensitive nanocarriers, which use the oxidative stress-associated microenvironments in cancer, inflammatory diseases, ischemia, and neurodegeneration. Through the integration of antioxidant modulation with site-specific drug or gene release, these platforms go beyond nonspecific scavenging and towards spatially and temporally controlled therapeutic intervention. Similarly, there have been many advances in lipid nanoparticles, polymeric systems, and inorganic carriers that have pushed gene delivery technologies for siRNA, mRNA, and CRISPR/Cas applications, while intracellular release strategies that are redox triggered improve cytosolic bioavailability and therapeutic precision. Theranostic nanoplatforms are also excellent examples of the combination of therapy and diagnostics, as they support the real-time imaging of biodistribution and treatment response while also delivering therapeutic payloads. Such an integration helps with adaptive, image-guided interventions and aligns well with the principles of precision medicine. Despite these developments, there are challenges surrounding its safety, long-term toxicity, manufacturing reproducibility, and regulatory acceptance, all of which remain as barriers to its widespread clinical translation. It will be necessary to address these issues through standardized characterization, adaptable production methods, and collaborative regulatory systems. Ultimately, the continued convergence of material science, redox biology, genetic engineering, and imaging technologies places nanomedicine at the forefront of next-generation, patient-specific therapeutic strategies.

## Data Availability

No new data were created or analyzed in this study. Data sharing is not applicable to this article.
